# Targeted testing for *DNAI1* hot spot-mutation utilizing immunofluorescence microscopy findings

**DOI:** 10.1186/2046-2530-1-S1-P104

**Published:** 2012-11-16

**Authors:** M Szczepaniak, NT Loges, H Olbrich, M Witt, H Omran

**Affiliations:** 1 Department of Clinical and Molecular Genetics, Institute of Human Genetics, Polish Academy of Sciences, Poznań, Poland; 2 International Institute of Molecular and Cell Biology in Warsaw, Poland; 3 Klinik und Poliklinik für Kinder- und Jugendmedizin –Allgemeine Pädiatrie-, Universitätsklinikum Münster, Germany

## 

Primary ciliary dyskinesia (PCD) is a rare (prevalence 1/20,000) genetic disease affecting motile cilia in the respiratory epithelium, spermatozoid flagella and primary cilia in the embryonic node. The most frequent (~60-70%) structural defect identified by TEM in the cilia of PCD patients are abnormal dynein arms. Several genes can cause PCD, but the majority of mutations were found in *DNAH5* and *DNAI1* genes (respectively ~28% and 4-10% of all cases), which encode heavy and intermediate chains of the outer dynein arms (ODAs), respectively. Mutations in both genes account collectively for almost 40% of PCD cases [Olbrich et al. 2002, Hornef et al. 2006, Zariwala et al. 2006, Zietkiewicz et al. 2010]. The hot-spot mutation in the *DNAI1* gene appears in intron 1, with the frequency of the most popular mutation (IVS1+2_3insT, causes aberrant splicing) around 55% of all *DNAI1* mutations. We have previously shown in a few PCD cases that proximal type-1 ODA complexes can be at least partially assembled in *DNAI1*– mutant cilia [Fliegauf et al. 2005]. We analysed the frequency of this hot spot mutation among 51 patients, in which immunofluorescence has identified abnormal ODA staining (proximal presence of DNAH5). The prevalence of the mutation in intron 1 of *DNAI1* gene will be confirmed by PCR and restriction enzyme digestion. In addition, we analyzed respiratory cilia for the inner dynein arm component DNALI1 localization, which we expect not to be altered in *DNAI1* mutant cilia, contrasting the findings present in *KTU*- mutant cilia.

